# Assessment of fetal growth and anomalies in the era of COVID-19 pandemic: an Egyptian pilot study

**DOI:** 10.1186/s43043-021-00075-2

**Published:** 2021-08-28

**Authors:** Emad Eltemamy, Sameh Salama, Sondos M. Salem, Mazen Abdel-Rasheed, Ehab Salama, Sherif Elsirgany, Tamer Elnahas

**Affiliations:** 1 Obstetrics and Gynecology Department, Faculty of Medicine, Al-Azhar University, Cairo, Egypt; 2 Reproductive Health and Family Planning Department, National Research Centre, Giza, Egypt

**Keywords:** Fetal growth, Anomalies, COVID-19 pandemic

## Abstract

**Background:**

Many issues need to be studied regarding pregnant women during the SARS-CoV-2 (COVID-19) infection pandemic. The aim of this study was to assess fetal growth, fetal well-being, and any observed gross anomalies that may follow SARS-CoV-2 infection in Egyptian pregnant women. During fetal anomaly scan at 22 weeks, we compared 30 pregnant women with a history of SARS-CoV-2 infection at 6‑12 weeks of gestation (group A) with 60 pregnant women (group B) who had no history of SARS-CoV-2. Then, we followed them on 28 and 34 weeks of gestation with fetal biometry and Doppler study.

**Results:**

Our results revealed no significant difference between both groups regarding fetal biometry, estimated fetal weight, amniotic fluid index, Doppler scan, and gross anomaly scan throughout all visits.

**Conclusion:**

According to the results of our pilot study, SARS-CoV-2 infection in pregnancy was not found to increase the risk of fetal growth restriction or possible fetal gross anomalies. Nevertheless, larger-scale studies are needed to confirm those findings. Perhaps, post-SARS-CoV-2 infection pregnancies may run an uncomplicated course regarding fetal parameters.

## Background

By the end of 2019, four cases of pneumonia of unknown etiology were reported to World Health Organization (WHO). Wuhan, China, was the first affected place worldwide by the SARS-CoV-2 infection [1]. In March 2020, WHO had raised up SARS-CoV-2 infection to the mark “pandemic” [2].

SARS-CoV-2 is a capsulated single-stranded RNA virus [3]. The known mode of transmission is droplet infection, close contact between people, and dealing with infected person stuff. The virus enters the body through the respiratory tract to affect pulmonary cells [4]. Pyroptosis, programmed cell death due to inflammation, of the pulmonary cell is the final destination of the cell [5]. A “cytokine storm” was also reported with SARS-CoV-2 infection that may lead to severe conditions and multisystem organ failure associated with high morbidity and mortality [6].

Fever, cough, lymphopenia, and elevated C-reactive protein levels are the main diagnostic signs of SARS-CoV-2 infection in pregnancy [7]. There are no studies that confirmed that pregnant women are at risk of infection more than the rest of the population due to pregnancy itself, rather than other factors that can affect general population health, as ethnicity. A recent American study found that the SARS-CoV-2 infection rate was more among pregnant women than adults of the same age from both genders. After excluding pregnant women with asymptomatic SARS-CoV-2 infection discovered before delivery, the results remained the same [8].

A higher rate of thromboembolic complications was observed in SARS-CoV-2 infection cases in the general population. Therefore, the activation of coagulation pathways followed by the possibility of developing vascular coagulopathy (DIC) as well as thrombocytopenia could be the most compelling explanation till now [9].

Pregnancy being known as a hypercoagulable state that is characterized by an increase in thrombin production and an increase in intravascular inflammation. The higher levels of coagulation and fibrinolytic factors, as plasmin, that are circulating in the blood of pregnant women may play a role in the pathogenesis of SARS-CoV-2 infection among pregnant women [10]. Mortality due to thromboembolic issues is higher with pregnant women than nonpregnant ones [11]. This additive risk of thrombosis urges researchers to recommend thromboprophylaxis until 10 days postnatal [12].

Pathological examination of the placentae from SARS-CoV-2 women had shown frequently increased fibrinoid deposition, enhanced inflammation, or maternal vascular malperfusion (MVM), including thrombi. All these findings are indicators for placental injury, which in turn lead to fetal distress with possible newborn long-term effects. Pathologic maternal blood flow due to MVM can lead to abnormal oxygenation with the possibility of adverse fetal outcomes like fetal demise [13]. Although a higher rate of pregnancy complications due to viral infections existed, there is still no definitive proof of post-SARS-CoV-2 consequences [14].

## Methods

This pilot case-control study was carried out on 30 pregnant women between 22 and 34 weeks of pregnancy who had positive PCR nasopharyngeal swab for SARS-CoV-2 infection between 6 and 12 weeks of pregnancy, followed by a negative swab to confirm recovery. They were assigned to group A, the study group (*n* = 30), and were compared to other 60 pregnant women with no history of SARS-CoV-2 infection during their first-trimester group B (*n* = 60). All participants were recruited from the antenatal outpatient clinics in the Medical Research Centre of Excellence (National Research Centre), Faculty of Medicine (Al-Azhar University), over a period of 8 months from August 2020 till March 2021. Ethical approval was taken from the ethical committee, Faculty of Medicine, Al-Azhar University (no. 00000430), before starting the study. Written consent was taken from all participated women in the study after a proper explanation of the aim and design of the study.

All women included in the study fulfilled the following criteria: age 20‑40 years, BMI 20‑30 kg/m^2^, singleton pregnancy, and first trimesteric scan including nuchal translucency assessment between 11 and 14 weeks of pregnancy as screening for possible chromosomal diseases. In addition, group A included only cases of mild and moderate SARS-CoV-2 infection, who did not need hospitalization or oxygen support, occurred between 6 and 12 weeks of pregnancy and confirmed by a positive nasopharyngeal swab using PCR technique followed by a negative PCR swab after recovery.

Women with any of the following criteria were excluded from the study; severe cases of SARS-CoV-2 infection that required antiviral, corticosteroids, or respiratory assistance, multiple pregnancies, smoking and medical disorders such as diabetes, hypertension, antiphospholipid, or thyroid diseases, using an anticoagulant treatment for any reason, cases taking any medications that could affect the fetus as anti-epileptics, retinoids, corticosteroids, cases who had been diagnosed, during the first trimester, with other infections that could affect the fetus as parvovirus, cytomegalovirus, rubella, or toxoplasmosis.

Gestational age was calculated using the first day of the last menstrual period if women were sure of their dates and had regular cycles for 3 months before conception. This was confirmed by 1st trimester dating ultrasound scan.

All women were subjected to a second-trimester fetal anomaly scan at 22 weeks with uterine artery Doppler (calculated from the reported last menstrual period or adjusted to the first-trimester crown-rump length measurement) and followed up at 28 and 34 weeks by ultrasound and Doppler study to monitor fetal growth, amniotic fluid, and fetal blood supply through measuring umbilical blood flow (umbilical PI) and evaluation of diastolic flow, measuring middle cerebral artery (MCA) flow and ductus venosus, if there was any growth restriction noted during flow up.

Also, a routine obstetric antenatal assessment was done in each visit for all women: measuring blood pressure, pulse, respiratory rate, weight, general examination, and abdominal examination. Obstetric examination was done to assess fundal level in correlation to the last menstrual period. Routine antenatal laboratory investigations were done as scheduled.

### Ultrasound studies

The fetal anomaly scan and Doppler studies at 22 weeks were done for all patients by a professional fetal medicine consultant using ultrasound machine GE Voluson P8 with RAB 2-6 RS Real-time convex transducer 2–5 MHZ (Chicago, IL, USA). According to the International Society of Ultrasound in Obstetrics and Gynecology (ISUOG) Guidelines in 2010, the following sonographic parameters were used to estimate fetal weight and size: biparietal diameter (BPD), head circumference (HC), abdominal circumference (AC), and femur diaphysis length (FDL), where we used the Astraia software (copyright 2000‑2009, version 1.20.0 Build 139) for data entry and reporting.

- Biparietal diameter (BPD): Caliper placement using outer edge to inner edge, at the widest part of the skull, using an angle perpendicular to the midline falx.
- Head circumference (HC): Caliper placed around the outside of the fetal skull bone echoes by using an ellipse.
- Abdominal circumference (AC): Caliper placed at the outer surface of the skin line and two linear perpendicular measurements, usually the anteroposterior abdominal diameter (APAD) and transverse abdominal diameter (TAD) taken.
- Femur diaphysis length (FDL): Caliper placed at the ends of the ossified diaphysis without including the distal femoral epiphysis if visible.
- Uterine artery Doppler technique: Transabdominally, the probe was placed longitudinally in the lower lateral quadrant of the abdomen, angled medially. Color flow mapping was helpful to identify the uterine artery as it was seen crossing the external iliac artery. The sample volume is placed 1 cm downstream from this crossover point.
- Umbilical artery Doppler: A free-floating cord was determined, and the sample gate volume was placed over it, measuring 1 cm (two-third over the artery and one-third over the vein). The angle between the ultrasound beam and the direction of blood flow was kept as close as possible to 0°. Measuring 3‑6 waves in the image with avoiding any fetal movements or breathing movements during measurements.

### Statistical analysis

Statistical analyses were performed using the SPSS software (SPSS, version 25, SPSS, Inc., IL, USA). The distribution of the measured variables was determined using the Shapiro test. Normally distributed variables were presented as means ± standard deviation (SD, statistical significance of differences for normally distributed variables was tested using the Student’s *t* test), while categorical data differences were compared by the Chi-square test. For all statistical tests, *p* values were considered statistically significant if less than 0.05.

## Results

We studied 90 pregnant women divided into two groups. Women in group A (*n* = 30), who had a history of confirmed SARS-CoV-2 infection during their first trimester, were compared with those in group B (*n* = 60) who had no history of SARS-CoV-2 infection. When comparing the demographic characteristics for both groups, including age (*p =* 0.825), BMI (*p =* 0.659), parity (*p* = 0.865), no significant differences were detected (Table 1).

**Table 1:** Demographic characteristics of pregnant women in both groups

	“Group A” (***n*** = 30)	“Group B” (***n*** = 60)	***P*** value
**Age (years)**	25.77 ± 2.62	25.90 ± 2.72	0.825
**BMI (kg/m**^**2**^**)**	25.84 ± 2.98	25.58 ± 2.54	0.659
**Parity**	1.87 ± 0.90	1.90 ± 0.86	0.865
**GA at SARS-COV-2 infection**	9.37 ± 2.09	NA	NA
**GA at SARS-COV-2 recovery**	11.83 ± 2.15	NA	NA

All women who participated in the study had a second trimesteric anomaly scan at 22 weeks of pregnancy. Concerning the fetal biometric measurements (BPD, HC, FL, AC), estimated fetal weight (EFW), no significant difference was found. In addition, the same insignificant values were found between both groups regarding uterine artery Doppler scan (mean uterine artery PI), amniotic fluid had done at 22 weeks (Table 2).

**Table 2:** Fetal assessment at 22 weeks of gestation in both groups

	“Group A” (***n*** = 30)	“Group B” (***n*** = 60)	***P*** value
**BPD (22 weeks)**	56.93 ± 2.84	57.43 ± 2.94	0.444
**HC (22 weeks)**	197.83 ± 9.65	200.67 ± 9.87	0.199
**FL (22 weeks)**	36.63 ± 2.57	37.15 ± 2.84	0.403
**AC (22 weeks)**	169.53 ± 12.16	169.05 ± 10.48	0.846
**EFW (22 weeks)**	473.89 ± 56.22	479.43 ± 56.05	0.660
**Mean uterine PI (22 weeks)**	1.07 ± 0.22	1.05 ± 0.22	0.766
**Amniotic fluid: Average**	30 (100%)	60 (100%)	NA
**Gross anomalies**	1 (3.33%)	1 (1.67%)	0.613

In group A, we did not encounter any case with gross fetal anomalies except one case with an echogenic focus in the left ventricle, which could be considered a normal finding in the minority of fetuses. While in group B, a single case with fetal cleft lip and cleft palate was diagnosed by the ultrasound with no other structural anomalies.

At the 28 weeks scan, we had only one case of increased AFI > 95th percentile for gestational age (polyhydramnios) in group B. When ultrasound was done to all cases at 34 weeks, a case in group A had a fetus showed an estimated fetal weight on the 3rd centile, BPD on the 15th centile, and AFI of 5 with preserved normal Doppler parameters, picture suggestive of asymmetrical fetal growth retardation. The woman in group B, who had been diagnosed with polyhydramnios at 28 weeks, continued to have the same picture at 34 weeks ultrasound. Her investigations were all normal (idiopathic polyhydramnios). Another two cases in the same group showed an ultrasound picture of oligohydramnios, AFI < 5th percentile for age, with preserved normal fetal growth pattern. One of them was diagnosed with a confirmed rupture of membranes, temperature monitoring, and investigations (TLC, CRP) and then was managed accordingly. In the other case, we did not find an explanation for the decreased amniotic fluid (idiopathic oligohydramnios).

Accordingly, there was no statistically significant difference between both groups regarding the fetal biometry (BPD, HC, FL, AC), estimated fetal weight, uterine Doppler (mean uterine artery PI), amniotic fluid index, gross anomalies, and the umbilical artery PI (Tables 3, 4).

**Table 3:** Fetal assessment at 28 weeks of gestation in both groups

	“Group A” (***n*** = 30)	“Group B” (***n*** = 60)	***P*** value
**BPD (28 weeks)**	75.07 ± 3.61	75.03 ± 3.56	0.967
**HC (28 weeks)**	261.67 ± 10.19	263.83 ± 10.32	0.348
**FL (28 weeks)**	52.37 ± 2.74	52.38 ± 2.77	0.979
**AC (28 weeks)**	235.77 ± 11.49	232.82 ± 15.29	0.309
**EFW (28 weeks)**	1205.33 ± 112.68	1187.48 ± 144.86	0.556
**Mean uterine PI (28 weeks)**	0.84 ± 0.15	0.85 ± 0.19	0.723
**Umbilical PI (28 weeks)**	1.11 ± 0.19	1.15 ± 0.20	0.333
**Amniotic fluid index**	13.67 ± 1.67	13.97 ± 2.33	0.531
**Gross anomalies**	1 (3.33%)	1 (1.67%)	0.613

**Table 4:** Fetal assessment at 34 weeks of gestation in both groups

	“Group A” (***n*** = 30)	“Group B” (***n*** = 60)	***P*** value
**BPD (34 weeks)**	89.80 ± 3.93	88.52 ± 3.72	0.133
**HC (34 weeks)**	311.17 ± 13.30	308.27 ± 11.54	0.289
**FL (34 weeks)**	64.23 ± 2.92	64.93 ± 3.16	0.312
**AC (34 weeks)**	291.37 ± 18.10	287.32 ± 15.60	0.274
**EFW (34 weeks)**	2277.84 ± 260.01	2234.54 ± 233.58	0.427
**Mean uterine PI (34 weeks)**	0.79 ± 0.14	0.73 ± 0.16	0.118
**Umbilical PI (34 weeks)**	0.92 ± 0.17	0.93 ± 0.17	0.777
**Amniotic fluid index**	11.4 ± 1.99	11.5 ± 3.13	0.874
**Gross anomalies**	1 (3.33%)	1 (1.67%)	0.613

## Discussion

Since there is no clear evidence to confirm or rule out an association between ethnicity and outcome in SARS-CoV-2 [15], every country must study the effect of SARS-CoV-2 on their populations at different health conditions. Emeruwa and his coworker found that Hispanic pregnant women are more susceptible to severe SARS-CoV-2 infection and worse pregnancy outcomes [16]. It is expected that infection by the SARS-CoV-2 virus during pregnancy may increase the risk of maternal and fetal health complications [17]**.** As SARS-CoV-2 is still a new disease with limited experience worldwide, it is still early to draw evidence about an increased risk of severe consequences of SARS-CoV-2 infection on pregnant women.

This pilot study was done to address possible consequences of post-COVID infection on fetuses of 30 Egyptian women who had been infected with SARS-CoV-2 infection between 6 to 12 weeks of pregnancy. After confirmation of their recovery by negative PCR test, anomaly scan was done at 22 weeks, and antenatal follow-up with repeated growth scans and Doppler were done sequentially at 28 and 34 weeks. After analyzing our results, we found that both groups were comparable in all parameters studied with no significant findings.

At the 22 weeks ultrasound, only one case in group A was found to have an echogenic focus in the left ventricle, which could be considered a normal variant. An isolated echogenic focus in the fetal heart at mid-trimester ultrasound scan in women aged 18–34 years is not associated with increased risk for trisomy 21 [18]. As our patient was 28 years old, no amniocentesis was done after explanation to the patient. Also, this patient had a first-trimester scan with normal nuchal translucency measurement. Another case in group B was diagnosed with cleft lip and cleft palate without any other gross anomalies, which is the most common fetal craniofacial malformation that is screened during prenatal ultrasonographic examination [19]. Cosma’s research group found that asymptomatic or mildly symptomatic SARS-CoV-2 infection during the first trimester of pregnancy did not predispose affected women to more fetal anomalies than unaffected women [20], and Rosen and his coworkers also confirmed the same results on the first and second trimester with no significant adverse neonatal outcome [21].

When we monitored all the women in both groups regarding growth parameters and Doppler studies at 22, 28, and 34 weeks, we observed that pregnancies complicated by SARS-CoV-2 infection had no significant change in fetal growth parameter, velocity, and fetal Doppler compared to pregnancies not complicated SARS-CoV-2 infection. American research that addressed this issue very close to our goal was published early this year. They concentrated on fetal growth and hemodynamics. After studying 49 cases complicated by mild SARS-CoV-2 infection and a 98 case not complicated with the viral infection, in agreement with our study, they did not find any significant difference between post viral infection group and the control group [22]. They did not include moderate cases of SARS-CoV-2 infection, which in our opinion could be the degree of disease that can have a real impact on pregnancy due to the disease itself and not due to the sequelae of the disease. The mild form of the disease can be due to good immunity that can diminish the effect of the disease.

On the contrary, Anuk and his team found that the PI and RI of uterine and umbilical artery increased significantly in pregnant women in the SARS-CoV-2 infection group compared to the control group (*p* < 0.05). Although the pathophysiology of SARS-CoV-2 infection in pregnancy has not been absolutely clear, they considered that third-trimester uterine artery measurement with biometric measures is enough to predict the risk of late-onset fetal growth restriction and stillbirth. However, they only found one case of intrauterine growth restriction in the study group [23]**.** These controversial results could be due to the difference in the degree of infection included as they include severe cases in their study. In our opinion, severe cases have different factors that may affect the study results as the severe form of the disease is associated with secondary infections and different organ function affection, which in turn can mislead the results.

At 34 weeks, we recorded a case in group A diagnosed with asymmetrical fetal growth restriction (EFW < 3rd centile) and oligohydramnios (AFI = 5) with normal Doppler parameters. However, this might have been related to a history of previous preterm delivery with fetal growth restriction in her first pregnancy.

Most of the researchers that found intrauterine growth restriction was between case reports and studies that recommend larger sample size studies [24, 25], while others did not find any growth restriction [26, 22].

Although some papers talked about maternal-fetal placenta perfusion affection, they could not confirm fetal growth restriction [27]. A higher rate of malperfusion signs has been found in the SARS-CoV-2 positive placentas, such as villous agglutination, microcalcifications, syncytial knotting, and fibrin thrombi. However, there was no negative impact on other variables as coagulation or inflammation, and in turn, there was no rise in the worse fetal outcome as IUGR [28]**.**

We searched in the literature, and we found that, in Egypt, a very limited number of studies had been done on the possible detrimental effects of SARS-CoV-2 on fetuses when infection acquired during early pregnancy. Thus, we hope that this pilot study will be a cornerstone for further larger multi-centric studies.

Despite that, we have some limitations in our study; the short follow-up period and failure to complete the antenatal care after 34 weeks until the delivery date was one of our limitations in the study, which could lead to underestimation of risk fetal growth restriction. The socioeconomic characteristics and psychological stress during pregnancy can affect our results. We found great difficulty in collecting cases included in the study group due to the poor registry system for cases of SARS-CoV-2 infection, especially mild and moderate cases, as they usually received their medical care at home. We included only patients with the laboratory-confirmed positive qRT-PCR assay. However, the viral nucleic acid test has a false-negative rate of up to 30% [29].

## Conclusions

It is difficult to draw conclusions from a small-scale pilot study. However, our results found out that SARS-CoV-2 infection during early pregnancy did not have an impact on gross anomalies or fetal growth. In turn, SARS-CoV-2 infection during early pregnancy may not pose a risk on fetal intrauterine well-being. The impact of SARS-CoV-2 in pregnancy remains to be determined, and a concerted, global effort is required to determine the effects on implantation, fetal growth and development, labor, and neonatal health.

## Data Availability

The datasets generated during and/or analyzed during the current study are available from the corresponding author on reasonable request.

## Abbreviations

AC: Abdominal circumference
BPD: Biparital diameter
EFW: Estimate fetal weight
FDL: Femur diaphysis length
HC: Head circumference
PCR: Polymerase chain reaction
SARS-CoV-2: Severe acute respiratory syndrome coronavirus 2
